# *Williamsia muralis* bacteraemia in a patient with Fanconi anaemia after haematopoietic cell transplantation

**DOI:** 10.1099/acmi.0.000679.v3

**Published:** 2023-12-04

**Authors:** Motoshi Sonoda, Yoshitomo Motomura, Masataka Ishimura, Shunsuke Kanno, Makiko Kiyosuke, Shouichi Ohga

**Affiliations:** ^1^​ Department of Pediatrics, Graduate School of Medical Sciences, Kyushu University, Fukuoka, Japan; ^2^​ Department of Pediatrics, National Hospital Organization Kyushu Medical Center, Fukuoka, Japan; ^3^​ Department of Clinical Chemistry and Laboratory Medicine, Kyushu University Hospital, Fukuoka, Japan

**Keywords:** 16S rRNA sequencing, bacteraemia, catheter-related bloodstream infection, haematopoietic cell transplantation, *Williamsia muralis*

## Abstract

**Introduction.:**

*
Williamsia muralis* is an environmental bacterium first detected in 1999. Infections with *W. muralis* isolated have been reported in two elderly patients, and were associated with the surgical intervention of artificial objects. We present a case of bacteraemia caused by *W. muralis* following haematopoietic cell transplantation (HCT).

**Case presentation.:**

A 10-year-old Japanese boy presented with fever and the swelling of the left cheek 8 days after HCT for the treatment of Fanconi anaemia. Gram-positive, rod-shaped bacteria were isolated from the blood cultures after 5 days incubation. 16S rRNA sequencing, but not mass spectrometry, identified a strain of *W. muralis* (1 414 bp, %ID 100 %). The phlegmon did not respond to antimicrobial therapy, but remitted with defervescence after a successful engraftment with teicoplanin and meropenem therapy on day 16 after HCT. The patient experienced recurrence of the bacteraemia, leading to central venous catheter (CVC) line removal. The same strain of *W. muralis* was isolated from the cultured tip of the CVC. To our knowledge, this is the first reported case of *W. muralis* bacteraemia and was complicated by CVC infection after HCT.

**Conclusion.:**

*
W. muralis* bacteraemia developed in an immunocompromised child. Introduction of artificial objects into the body raises a risk of rare infection with slowly growing environmental bacteria.

## Data Summary

No data was generated or reused during this research.

## Introduction

*Williamsia muralis* is a slow-growing, aerobic, Gram-positive rod bacteria belonging to the family *Nocardiaceae* [1]. It was first isolated in 1999 and later classified as an environmental bacterium within the genus *Williamsia *. Twelve species of *Williamsia* have been reported to date, including *Williamsia aurantiacus *, *Williamsia deligens *, *Williamsia faeni *, *Williamsia herbipolensis *, *Williamsia limnetica *, *Williamsia maris *, *Williamsia marianensis *, *W. muralis *, *Williamsia phyllosphaerae *, *Williamsia serinedens *, *Williamsia spongiae* and *Williamsia sterculiae* [2].

Limited information is available about human infection with *Williamsia* spp. because only a few cases of isolated infection have been reported in elderly patients [3]. It appeared that *W. muralis* infection was associated with surgical intervention/prosthetic material. We herein present the first case, to our knowledge, of *W. muralis* bacteraemia, which occurred in a paediatric patient after haematopoietic cell transplantation (HCT).

## Case presentation

### Clinical findings

**Fig. 1.**
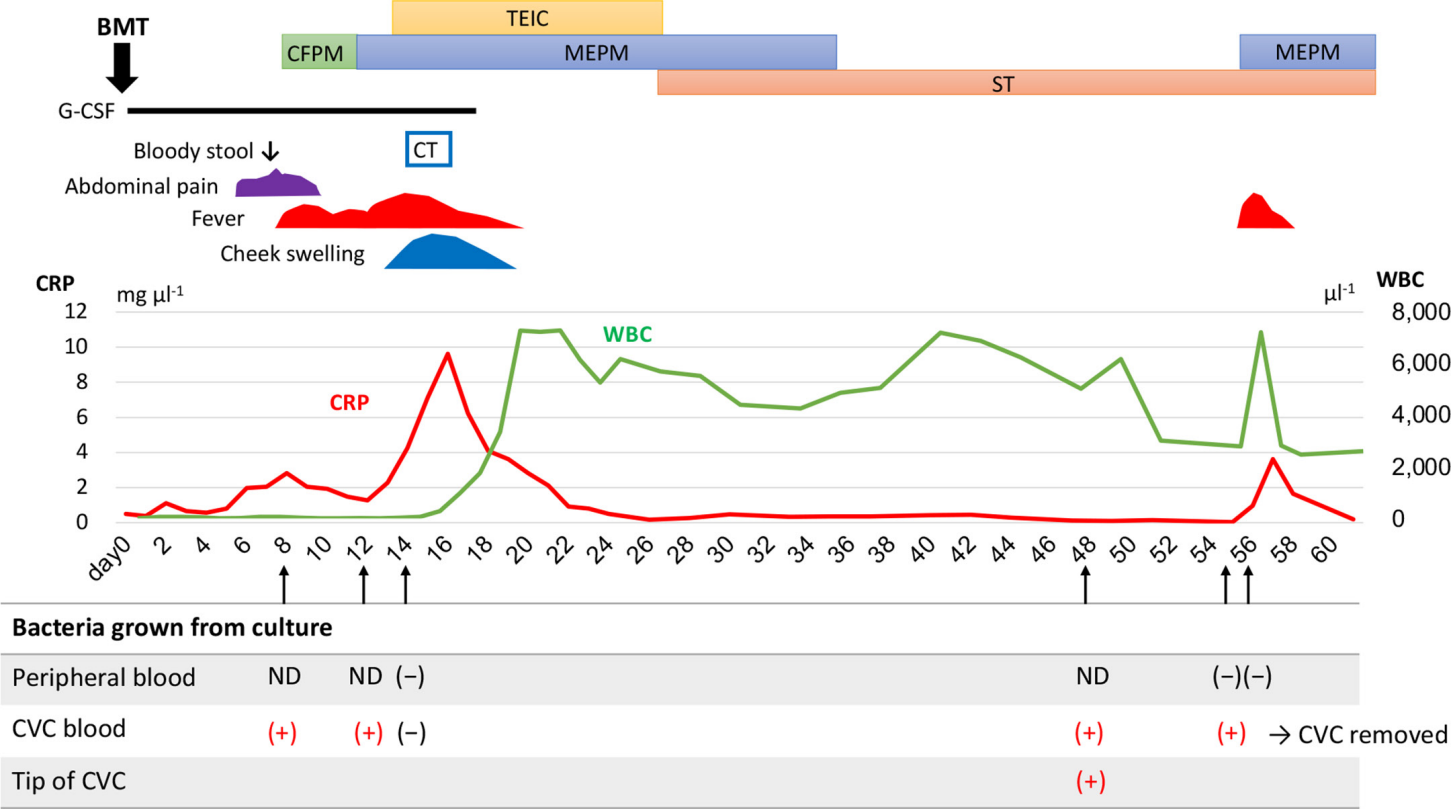
Clinical course after bone marrow transplantation in a patient with *W. muralis* detected . BMT, Bone marrow transplantation; CFPM, cefepime; CRP, C-reactive protein; CT, computed tomography; G-CSF, granulocyte colony stimulating factor; MEPM, meropenem; ND, not done; ST, sulfamethoxazole/trimethoprim; TEIC, teicoplanin; WBC, white blood cell.

**Fig. 2.**
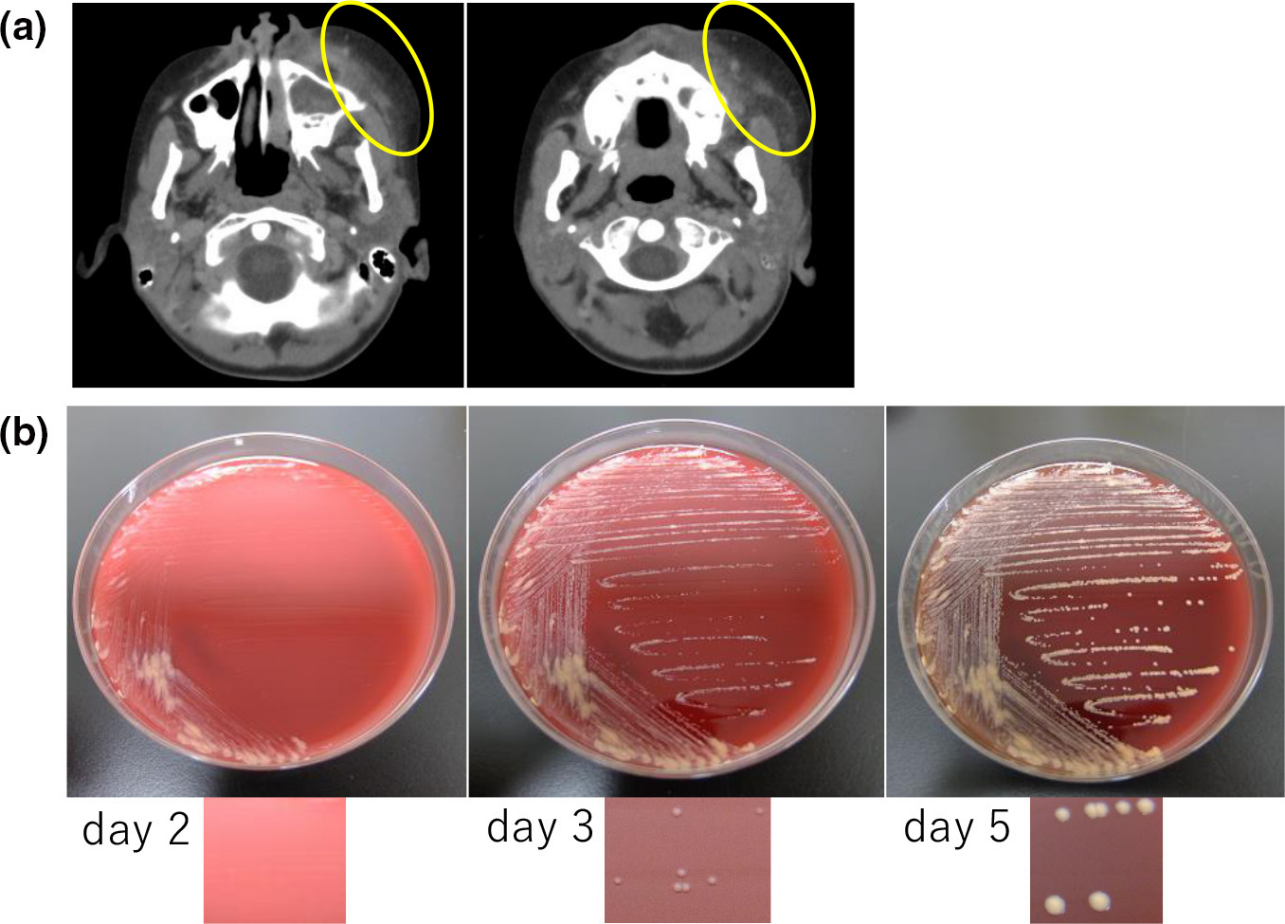
(a). Facial cellulitis on the patient’s left cheek (yellow circles). (b). Blood culture results over time. Gram-positive, rod-shaped bacteria were isolated from the blood culture samples after 5 days of incubation. Small colonies were observed after 2 days of incubation on 10 % sheep blood agar plates (left picture). The colonies were round, pale orange, Gram-positive bacilli, and their diameter expanded to 2–3 mm after 5 days of additional incubation (middle and right pictures).

A 10-year-old Japanese boy with transfusion-dependent Fanconi anaemia underwent HCT. On day 8 after HCT, the patient complained of abdominal pain with vomiting and bloody stool (Fig. 1). His body temperature was 38 °C. The laboratory findings revealed a white blood cell count of 40 cells µl^−1^ and an elevated C-reactive protein concentration of 2.81 mg µl^−1^. For the control of post-transplant febrile neutropenia, cefepime 50 mg kg^−1^, twice daily, was administered after the first blood culture from the central venous catheter (CVC). Because of persistent fever for 4 days, the antibacterial agent was switched to meropenem, 100 mg kg^−1^ per day for 24 days, after the second blood culture. His left cheek became swollen on day 14 after HCT. Enhanced computer tomography findings led to a diagnosis of facial cellulitis (Fig. 2a).

### Investigations and diagnosis

On microbiological analysis, Peds Plus (PED) and Plus Anaerobic culture vials (Becton Dickinson; BD) were used in a BD BACTEC FX system. Gram-positive, rod-shaped bacteria were isolated from the first two blood cultures in PED vials after a 5 day incubation period. Both blood culture-positive samples were collected from the CVC. Small colonies were observed after incubation for 48 h at 35 °C on 5 % sheep blood agar plates (Shimazu) in a 5 % CO_2_ environment. These colonies appeared as round, pale orange, Gram-positive bacilli that expanded to a diameter of 2–3 mm after 5 days of additional incubation (Fig. 2b). The isolated bacteria were catalase-positive, but negative for Kinyoun’s acid-fast staining. However, it was difficult to identifying the Gram-positive bacilli biochemically because of disparate results between VITEK 2 ANC and API Coryne tests. The VITEK2 ANC system identified the bacilli as *Corynebacterium urealyticum* (% ID: 96 %), while the API Coryne test after 24 h of incubation indicated that the bacilli were *Rhodococcus* spp. (% ID: 94.7 %). VITEK MS, an automated mass spectrometry using matrix-assisted laser desorption/ionization-time of flight (MALDI-TOF) technology, didn’t identify the strain either. Because of the failure to determine the strain using these devices and systems, we performed 16S rRNA sequencing with an ABI 3500xL automated DNA sequencer (Applied Biosystems) under the guidelines of the Clinical and Laboratory Standards Institute (CLSI) (MM18-A) [4]. The sequence obtained had a continuous stretch of 1 414 bp that exhibited 100 % similarity to *W. muralis* in a previous report [1]. The antimicrobial-drug susceptibility of the isolated colony was determined using the agar plate dilution method. The isolate was incubated on microplates at 35 °C in an aerobic atmosphere for 72 h. The minimum inhibitory concentration of *W. muralis* was estimated as described by CLSI document M24-A2 [5], and susceptibility to several antibiotics, including meropenem and teicoplanin, was observed (Table 1).

**Table 1.** Antibiotic-susceptibility testing and interpretation for the *W. muralis* isolate

Drug	MIC (μg ml^−1^)	Category
Amoxicillin/clavulanate	≦2/1	S
Cefotaxime	1	S
Cefepime	≦0.5	S
Imipenem/cilastatin	≦0.25	S
Meropenem	≦0.25	–
Vancomycin	≦0.5	–
Teicoplanin	≦0.5	–
Linezolid	1	S
Amikacin	≦1	S
Tobramycin	≦0.25	S
Minocycline	1	S
Sulfamethoxazole/trimethoprim	≦9.5/0.5	S
Ciprofloxacin	0.25	S

MIC, Minimum inhibitory concentration; S, susceptible.

### Treatment and outcome

Three days after an additional administration of teicoplanin (initially 10 mg kg^−1^ every 12 hours for 3 doses, then 10 mg kg^−1^ once daily for 14 days), fever, C-reactive protein levels and cheek swelling peaked out on day 16 after HCT, coinciding with engraftment (i.e. white blood cells, 1010 cells µl^−1^, and neutrophils, 900 cells µl^−1^). A prompt improvement of fever and cellulitis was observed by day 18 after HCT (Fig. 1).

The post-transplant management required the persistent insertion of a CVC after HCT. Based on the result that the blood culture from the CVC on day 14 was sterile, antibiotic therapy was completed (23 days for meropenem and 15 days for teicoplanin). However, fever recurred on day 48 after HCT. The blood culture from the CVC was positive again for *W. muralis *, although the peripheral venous blood cultures were negative on day 48 and day 55 post-transplant. Under the diagnosis of catheter-related bloodstream infection, the CVC was removed. *W. muralis* was isolated again from the culture of the CVC tip. Meropenem, 100 mg kg^−1^ per day for 7 days, therapy was terminated 1 week after CVC removal, and no fever recurred thereafter.

## Discussion

We have described a case of *W. muralis* infection in an immunocompromised child who presented recalcitrant bacteraemia complicated by CVC infection. This is, to our knowledge, the first report of *W. muralis* bacteraemia, because the two previous cases were reported as having isolated infection with *W. muralis* presenting with endophthalmitis and pneumonia [6, 7].

We compared this rare human infection between the current case and the two reported cases (Table 2). *W. muralis* infection developed after the insertion of artificial objects during surgery, intraocular treatment or central venous nutrition. These findings alert that *W. muralis* infection occurs when environmental bacteria attach to these artificial objects. The three cases also indicate that *W. muralis* has a pathogenic potential in immunocompromised hosts. In the reported cases, local lesions developed within a few days after surgery. The first patient without immunodeficiency died of multiple organ failure from pneumonia. The short incubation time of the slowly growing bacteria indicated a property of the strain, otherwise a systemic infection occurring from the greater amount of contamination in the aged patient. Nevertheless, there was no description of the blood cultures in the deceased case. The facial cellulitis in the current case was regrettably not confirmed as bacteraemia origin because no punctures of the affected lesion were performed. Similarly, it was difficult to determine that the preceding bloody diarrhoea was induced by bacteraemia of *W. muralis* without identification in stool culture. We should also remark on the difficulty of laboratory identification of this organism and the risk of misidentification by commercially available biochemical systems. It is unclear whether the environmental bacteria of *W. muralis* form biofilms in the host. In this case, there was recalcitrant bacteraemia prior to line removal and positive culture from the line tip. *W. muralis* was considered to have enough time to form biofilm on the CVC tip during the 28 days after the insertion and during the conditioning phase of HCT. These findings could provide evidence that the environmental bacteria of *W. muralis* form biofilm on inserted artificial objects.

**Table 2.** Clinical presentation of three patients who developed *W. muralis* infection

Characteristic	del Mar Tomas (2005) [7]	Murray (2007) [6]	Present case
Diagnosis	Pulmonary infection	Endophthalmitis	Bacteraemia with facial cellulitis
Age (years)/sex	80/female	66/male	10/male
Background	Aortic stenosis	Maculopathy with diabetic mellitus	Fanconi anaemia
Event	48 h after aortic valve replacement	24 h after IVTA	28 days after CVC insertion (12 days after HCT)
Symptoms	Fever, respiratory impairment	Ophthalmalgia, reduced visual acuity	Fever, swelling on cheek
Specimen	Brush with bronchoscopy	Vitreous fluid	CVC blood, tip of CVC
Incubation	48 h	6 days	4–7 days (five specimens)
Detection (16S rRNA gene)	* W. muralis* (1438 bp, % ID 99%)	* W. muralis* (1428 bp, % ID 98.4 %)	* W. muralis* (1414 bp, % ID 100 %)
Antibiotics	1st: AMK, TEIC, LVFX	lntravitreal VCM and CAZ	1st: CFPM
	2nd: AMK, TEIC, IPM, FLCZ		2nd: MEPM, TEIC
Outcome	Died of MOF	Reduced visual acuity remained	Recurrent fever (CRBSI)

AMK, Amikacin; CAZ, ceftazidime; CFPM, cefepime; CRBSI, catheter-related bloodstream infection; FLCZ, fluconazole; ID, identification; IMP, imipenem; IVTA, intravitreal triamcinolone acetonide; LVFX, levofloxacin; MEPM, meropenem; MOF, multiple organ failure; TEIC, teicoplanin; VCM, vancomycin.

The clinical features of *W. muralis* infection share similarities with those of nocardiosis, occasionally causing a localized or disseminated infection in immunocompromised hosts. *Nocardia* spp. are environmental bacteria belonging to the same family (* Nocardiaceae *) as *W. muralis *. Nocardiosis particularly affects the skin, soft tissue and the nervous system through haematogenous dissemination. Unlike *Nocardia* spp., *W. muralis* might be susceptible to a wide range of antimicrobial drugs. Because there are no specific criteria for antimicrobial-drug susceptibility testing in *Williamsia* spp., we used the criteria for *Nocardia* spp., as described elsewhere [5]. The strain this patient contracted exhibited a wide spectrum of antimicrobial susceptibility. Further identification of cases of infection is awaited to determine an effective strategy of antimicrobial drugs and treatment duration.

### Conclusion

We report the first case, to our knowledge, of *W. muralis* bacteraemia in an immunocompromised host. The isolated or systemic infection with *W. muralis* suggested similarities with nocardial infections occurring after the insertion of artificial objects.
